# Effects on Cognition of Stereotactic Lesional Surgery For the Treatment of Tremor in Multiple Sclerosis

**DOI:** 10.3233/BEN-2008-0207

**Published:** 2009-06-01

**Authors:** Marjan Jahanshahi, Socorro Pieter, Sundus H. Alusi, Catherine R. G. Jones, Scott Glickman, John Stein, Tipu Aziz, Peter G. Bain

**Affiliations:** ^1^Sobell Department of Motor Neuroscience & Movement DisordersUCL Institute of NeurologyThe National Hospital for Neurology & NeurosurgeryLondonUK; ^2^The Walton Centre for Neurology and NeurosurgeryNHS TrustMerseysideLiverpoolUK; ^3^Current address: Behavioural and Brain Sciences UnitUCL Institute of Child HealthLondonUK; ^4^Current address: Buckinghamshire Chilterns University CollegeChalfont St. GilesBuckinghamshireUK; ^5^Department of PhysiologyAnatomy and GeneticsUniversity of OxfordOxfordUK; ^6^Department of NeurosurgeryRadcliffe InfirmaryOxfordUK; ^7^The Movement Disorders and Neurostimulation UnitDepartment of NeuroscienceCharing Cross Hospital and Division of Neuroscience and Mental HealthImperial College LondonLondonUK

**Keywords:** Multiple sclerosis, cognition, thalamotomy, surgery, tremor

## Abstract

*Objective:* To assess the effect of stereotactic lesional surgery for treatment of tremor in multiple sclerosis on cognition.

*Methods:* Eleven patients (3 males, 8 females) with multiple sclerosis participated in the study. Six subjects comprised the surgical group and five the matched control group. All patients were assessed at baseline and three months using a neuropsychological test battery that included measures of intellectual ability, memory, language, perception and executive function.

*Results:* There were no significant differences between the surgical and control groups and no change from pre to post testing except for a decline in scores on the Mini-Mental State Examination (MMSE), WAIS-R Digit Span and Verbal Fluency in the surgical group.

*Conclusions:* The results indicate that stereotactic lesional surgery does not result in major cognitive impairment in multiple sclerosis. However, the decline in MMSE scores, digit span and verbal fluency require further investigation in a larger sample.

## 1. Introduction

The thalamus has long been known as the major relay station modulating sensory signals to the cerebral cortex [27]. It is involved in a number of neural pathways, some of which affect aspects of cognition, such as language, attention, and memory [11]. Evidence for the involvement of the thalamus in cognition was presented by Van der Werf et al., who demonstrated that infarctions in the thalamic region lead to cognitive disturbance such as memory and executive deficits [55]. Similarly, lesions of the thalamus result in impaired cognitive function [1] and deficits in learning, memory and planning have been reported following lesions of the thalamus [9].

Lesioning of the thalamus or stereotactic thalamotomy has been found to be an effective treatment for movement disorders, particularly for the control of tremor [10,50]. Surgical lesioning of the thalamus has

ISSN 0953-4180/08/$17.00 © 2008 – IOS Press and the authors. All rights reserved

Table 1
The demographic and clinical details of the patients in the surgery and control groups

| Clinical features | MS surgery group | MS control group |
|---|---|---|
| Age (mean, years) | 46.3 ± 3.7 | 45.8 ± 4.2 |
| Sex ratio (male/female) | 2:4 | 1:4 |
| Disease duration (mean, years) | 16.5 ± 8.7 | 20.0 ± 5.8 |
| Age of onset (years) | 28.2 ± 11.5 | 25.8 ± 8.9 |
| EDSS (median) | 6.75 (IR 3.75–9.25) | 6.50 (IR 6.50–8.50) |
| Barthel ADL Index (median) | 7.75 (IR 5.38–11.25) | 16.00 (IR 4.00–16.50) |
| NART (estimate of premorbid IQ) | 92.50 ± 2.38 | 99.00 ± 11.90 |
| WAIS-R (current Verbal IQ) | 87.83 ± 12.77 | 88.6 ± 6.58 |
| MMSE (mean) | 26.33 ± 3.27 | 25.27 ± 1.96 |

EDSS: Expanded Disability Status Scale.
ADL: Activities of Daily Living.
NART: National Adult Reading Test.
WAIS-R: Wechsler Adult Intelligence Scale-Revised.
MMSE: Mini Mental State Examination.
IR: Interquartile Range.

been shown to influence cognitive function, particularly language functions and attention after left-sided thalamotomy in earlier reports [6,38,56]. With the resurgence of surgery for treatment of tremor in Multiple Sclerosis (MS), a number of more recent studies have also investigated the potential effects of this surgical procedure on cognition. In a randomized study comparing effects of thalamic stimulation and thalamotomy on patients with Parkinson's disease (PD), MS and essential tremor (ET), Schuurman et al., concluded that both procedures resulted in minimal overall risk of cognitive deterioration [46]. However, they found decreased Verbal Fluency following left-sided surgery. In contrast, Hugdahl and Wester concluded that stereotactic thalamotomy led to improvement of verbal memory in Parkinsonian patients [6]. In an earlier study by the same authors, Wester and Hughdal, there was no post-surgical deterioration in cognitive abilities in PD patients [61], which was also the conclusion arrived at by Fukuda et al. [17] and Maeshima et al. [24].

To date, studies investigating cognitive outcome after thalamotomy have mostly been on patients with PD. Although this procedure has also been used to treat tremor in MS since the 1960s, there is a dearth of neuropsychological data from this population. Cognitive deficits, particularly impairment of memory and attention are among the symptoms of MS [7,20,25,36,41], and it is possible that lesional surgery makes any preexisting cognitive problems worse or results in specific cognitive deficits following surgery. In their prospective case-controlled study, Alusi et al. [2] found that patients with multiple sclerosis who underwent stereotactic lesional surgery in the thalamic/subthalamic regions showed a non-significant post-operative decline in scores on the Mini-Mental State Examination. The current study was a more detailed investigation (i) to clarify whether thalamotomy/subthalamotomy produced any significant changes of cognitive function in a subgroup of MS patients from the study of Alusi et al. [2] and (ii) to identify the specific nature of any alterations of cognitive function following lesional surgery in MS.

## 2. Methods

Patients: Eleven patients (3 males, 8 females) with MS participated in the study. They fulfilled Poser's criteria for the clinical diagnosis of multiple sclerosis [35] and were assessed for suitability for surgery. These were 11 of the original 24 patients in the Alusi et al. [2] study for whom neuropsychological assessments were available both at baseline and three months after surgery. None of the patients were deliberately excluded or refused to participate in the neuropsychological assessment. For practical reasons, it was only possible to complete pre and post-operative neuropsychological assessments on 11 of the 24 patients.

Ethics approval was secured from the Riverside Ethics Committee and informed consent was obtained from the patients included in the study [2]. Six of the patients (2 males, 4 females) with a mean age 46.3 ± 3.7 years comprised the surgical group who underwent lesional surgery shortly after assessment at baseline. The average duration of illness for the surgical group was 16.5 ± 8.7 years. The other five patients (1 male, 4 females, 45.8 ± 4.2 years) comprised the control group and did not differ from the surgical group for age, age of onset of disease, Expanded Disability Status Scale (EDSS) [23], Barthel Activities of Daily Living (ADL)

Index [8], duration of illness, pre-morbid IQ (National Adult Reading Test, NART) [28], current estimates of Verbal IQ (Wechsler Adult Intelligence Scale–Revised, WAIS-R) [60], or global cognitive functioning assessed on the Mini-Mental State Examination (MMSE) [14], prior to surgery (Table 1). The average duration of illness for the control group was $20.0 \pm 5.8$ years. All but three (1 surgical and 2, non-surgical) of the participants in this study, were right-handed.

Design: A mixed design was used with Group (surgical versus control) as the between-groups factor and Time (baseline versus 3 months follow-up) as the within-subjects factor.

Surgical technique: Six patients underwent surgery for treatment of tremor. The details of the surgical procedures are provided in Alusi et al. [2]. Post-operative MRI confirmed that the lesion locations were in the left thalamus for three patients, in the left zona incerta extending to the thalamus in two and in the left subthalamic nucleus (STN) extending to the zona incerta in one.

Procedure: All surgical and control patients were assessed on two occasions: at baseline and three months later. They were assessed on the following battery of cognitive tests, selected to be sensitive to the types of deficits found in patients with MS [34].

### 2.1. *Tests of global cognitive functioning and intellectual ability*

1. Mini-Mental State Examination (MMSE) [14]. This short test is used to assess overall cognitive ability. It assesses various functions such as orientation (time and place), memory (registration and recall), working memory (attention and calculation), language (naming, repetition, reading and writing), visuo-spatial (copying of 2 overlapping pentagons), and executive function (3-stage command). The maximum score is 30. Scores below 23 are considered indicative of cognitive impairment.

2. Wechsler Adult Intelligence Scale-Revised (WAIS-R) [60]. This scale measures overall intellectual functioning. Five of the verbal subscales, Vocabulary, Similarities, Arithmetic, Comprehension and Digit Span, were administered to obtain a prorated estimate of current Verbal IQ.

3. National Adult Reading Test (NART) [28]. This test provides an estimate of pre-morbid IQ, based on the participants reading of 50 irregular nouns that can not be phonetically pronounced.

### 2.2. *Tests of memory, language and perception*

4. Rey Auditory Verbal Learning Test (RAVLT) [37]. This is a test of supraspan memory and verbal learning and retention. It consists of a list of 15 words that are read out five times. After each presentation, the subject is tested on free recall. Delayed recall (45 minutes) and delayed auditory recognition of the words among a list of 30 words containing 15 extra phonetically or semantically related words were also assessed. Performance on the first trial is a measure of immediate span. Performance across the 5 trials indicates the ability to learn the list. On each trial, the maximum score is 15.

5. Recognition Memory for Faces (RMF) [58]. This memory test consists of 50 photographs of men's faces, each presented for 3 s. Retention is tested immediately using a forced two-choice recognition format. The score is the total number of correct choices with a maximum of 50.

6. Subject-Ordered Pointing Test (SOPT) [31]. This is a test that requires holding and monitoring information 'on line' in working memory. This type of task was originally developed to show deficits after bilateral frontal lesions in monkeys [33] and subsequently was also shown to be sensitive to frontal pathology in humans [31]. In the present study, the stimuli were flags of African countries. Three versions of the task which differed in terms of the number of stimuli (4, 8 or 12) were used. In each condition, the subject was presented with a number of cards (either 4, 8 or 12) each bearing the same stimuli but arranged randomly on the card. The subject's task was to point to a stimulus on each card until all stimuli (either 4, 8 or 12) had been pointed to in turn. Two blocks of each of the three versions were performed. Selection of a stimulus more than once in a block of trials constituted an error. The mean number of errors across the two blocks was calculated for the 4, 8 and 12 versions.

7. Visual Object and Space Perception Test (VOSP) [59]. The Shape Detection and Fragmented Letters subsets of the VOSP battery were administered. The maximum score on each subtest is 20, with lower scores indicating greater perceptual difficulties.

8. Graded Naming Test (GNT) [26]. In this test, the participant is required to name 30 items from black and white line drawings, which are graded in increasing order of difficulty. The total number of items correctly named is a measure of the individual's current naming ability.

### 2.3. Tests of executive function and attention

9. Verbal Fluency (VF) [5]. In this test, participants are asked to generate words beginning with the letters F, A, S for 60 s each, excluding proper nouns, numbers and the same word with a different suffix. The score was the number of correctly generated words.

10. Cognitive Estimates Test (CET) [43]. This test is composed of ten questions designed to measure planning ability and the execution of an appropriate plan. An example of one of the questions is "What is the height of the Post Office Tower"? Patients with lesions of the prefrontal cortex perform poorly on this test [43].

11. Random Number Generation (RNG) [49]. Each participant was instructed to generate a series of 100 numbers in a random fashion. The analogy of picking numbers out of a hat was used to explain the concept of randomness to participants. Performance was paced with an auditory pacer presented at the rate of once every 2 s. The participants were instructed to synchronise their responses with the onset of the pacing tone. The total time taken to generate 100 items was recorded. The obtained measures of randomness were calculated using the procedures specified by Evans [13], Rosenberg et al. [39] and Ginsburg and Karpiuk [19].

(i) Repetitions (REP) measure the number of times the individual repeats the same item on successive trials. For example, 7-7 counts as 1 repeat, and 1-1-1 counts as 2 repeats.

(ii) Gap Score (GAP) is a measure of cycling through the set of 9 items. To obtain this measure, the gap between every occurrence of 1 is noted, then gaps between every occurrence of 2 are noted and so on, and the median is calculated. Higher scores indicate that the individual cycles through the series of 9 numbers in a regular fashion so that the numbers are too evenly spread out.

(iii) Count scores are measures of seriation. Count scores were obtained using the general method of Spatt & Goldenberg [47] Count Score 1 (CS1) measures the tendency to count in ascending or descending series in steps of 1, for example, 1-2-3 or 8-7-6-5-4. All count scores take the length of the series into account. In calculating the count scores, the sequence length is squared to give higher weights to runs of longer sequences. Therefore, these two examples would result in respective count scores of 4 (CS1 $= 2^2$) and 16 (CS1 $= 4^2$). Count Score 2 (CS2) measures the tendency to count in ascending or descending series in steps of 2, for example, 2-4-6-8 or 7-5-3-1. The Total Count Score (CST) is a composite measure of the individual's tendency to count in series ascending or descending in steps 1of 2. Individuals may have count scores that are lower than predicted from a random series if they are avoiding particular counting tendencies or they may have a score which is too high if they are unable to suppress particular counting tendencies.

(vi) Random Generation Index (RGI) is a first order measure which reflects any disproportion of diagrams in the matrix adjusted for disproportions in the marginal cell frequencies. It varies between 0 and 1, and the higher the index, the less random the series is.

Measures of randomness obtained from our sample were compared with similar measures calculated for computer-generated pseudo-random series. A sample of one hundred, 100-item series was generated using the algorithm RAN1 from Sprott et al. [51].

### 2.4. Assessment of effort and fatigue

Using a scale of 0 (no effort) to 10 (great effort), at the end of the assessment session, patients were asked to rate the amount of effort required to complete the tests. Patients were also asked to rate how tiring they found the assessment on a 0 (not tired at all) to 10 (very tired) scale.

Statistical analysis: Before data analysis, the Kolmogorov-Smirnov test was used to determine normality of the distribution for each variable. Skewness and kurtosis were also assessed. Where necessary, log transformations were used to normalize the data. A series of 2-way ANOVAs, with Group (surgery vs control) and Time (baseline vs 3 months) with repeated measures on the second factor were used. The level of significance for all analyses was set to 0.05. A Bonferroni correction was not applied to the statistical comparisons, because our aim was to investigate the full effects of surgery and therefore a low probability of Type II error was necessary [40].

## 3. Results

The current surgical patients were cases 3, 4, 5 , 7, 10, and 13 in the series reported by Alusi et al. [2]. The surgical group showed significant improvement of tremor ($F(1,5) = 46.24$, $p < 0.001$) at the 3 months post-op assessment (1.00 ± 1.14) compared to before surgery (7.67 ± 1.99). This improvement was evident in every case.

### 3.1. Global cognitive functioning and intellectual ability

The means and standard deviations of the cognitive tests administered to the surgical patients before and after surgery, and the control group on the two assessment occasions are presented in Table 2.

For the MMSE, the main effects of Group and Time were not significant ($p > 0.05$). There was a significant Group x Time interaction ($p = 0.034$) for the MMSE; with the simple effects analysis showing the surgery group scored higher at baseline than at 3 months ($p = 0.05$). Compared to pre-operative scores, all six patients in the surgery group had lower MMSE scores 3 months after surgery; which in three cases was below the cutoff score of 23 for cognitive impairment.

For the WAIS-R Verbal IQ, the main effects of Group and Time and the Group x Time interaction were not significant ($p > 0.05$). This was also true for the WAIS-R Vocabulary, Similarities and Comprehension subscales. For the WAIS-R Digit Span, a significant Time effect ($p = 0.003$) indicated that scores were lower at 3 months compared to baseline (3 months 7.36 ± 2.58 vs. baseline 8.64 ± 2.16). The Group x Time interaction was also significant ($p = 0.001$), which post-hoc analyses showed to be due to the surgery group scoring lower at 3 months compared to pre-operative baseline assessment ($p < 0.001$), whereas the control group showed no change from the first to second assessment ($p > 0.05$). This deterioration of Digit Span performance was observed in all 6 cases in the surgery group.

There was a significant main effect of Time ($p = 0.016$) for the WAIS-R Arithmetic subtest: the score at 3 months was *higher* than at baseline across the two groups (3 months 7.81 ± 2.63 vs. baseline 7.00 ± 2.86). There were no significant Group main effect ($p > 0.05$) or Group x Time interaction ($p > 0.05$) for the Arithmetic subtest.

### 3.2. Memory, language and perception

For the Rey Auditory Verbal Learning Test, the main effects of Group and Time and Group X Time interaction were not significant ($p > 0.05$). This was also true for the Self-Ordered Pointing Test, Recognition memory For Faces, Graded Naming Test and the Shape Detection or Incomplete Letters subtests of the VOSP battery ($p > 0.05$).

### 3.3. Executive function

There were no significant main or interaction effects for the Cognitive Estimates Test or any of the measures of randomness (repetitions, median gap index, count scores total and random generation index) obtained on the RNG test (all $p > 0.05$).

For the phonemic Verbal Fluency, the main effects of Group and Time were not significant.

However, there was a significant Group x Time interaction ($p = 0.035$). Further analysis showed that this was due to the surgical group generating significantly fewer words 3 months after surgery (15.00 ± 11.14) than at baseline (23.00 ± 12.90) ($p = 0.025$), whereas the control group showed no change from baseline (28.20 ± 9.36) to the 3 months (28.40 ± 6.95) assessment ($p > 0.05$). This worsening of verbal fluency after surgery was observed in all 6 operated cases.

### 3.4. Effort and fatigue

There were no significant main or interaction effects on the ratings of effort or fatigue ($p > 0.05$).

## 4. Discussion

Thalamotomy as a treatment for MS tremor has been used in the last four decades but studies investigating its effect on cognition are few, with research on the impact of this procedure mostly conducted on patients with PD. We undertook detailed neuropsychological assessment of the impact of stereotactic lesional surgery on cognition in MS and found that surgery did not result in any significant changes in intellectual ability, perception, or memory. The only significant changes from the pre to post-operative assessment were on verbal fluency, digit span and MMSE. Our results are consistent with those of Schuurman et al. [46] and the earlier report by Blumetti and Modesti [6] the two studies which included patients with multiple sclerosis assessed before and after thalamotomy. Schuurman et al. [46] compared the effects of thalamotomy and thalamic stimulation in 40 patients with Parkinson's disease (21 lesioning, 19 stimulation), 13 patients with essential tremor (6 lesioning, 7 stimulation) and 9 with multiple sclerosis (5 lesioning, 4 stimulation). They used a comprehensive battery of tests of cognitive function and found that only performance on the control reading tests of the Stroop and verbal fluency were significantly worse following surgery. They concluded that in MS thalamotomy is as-

Table 2
Means and standard deviation of the patients in the surgical and control groups on measures of global cognitive functioning, IQ, perception, memory and learning, language and executive function

| Measures | MS surgical patients | | MS control patients | |
|---|---|---|---|---|
| | Baseline | 3 months | Baseline | 3 months |
| MMSE* | 26.33 ± 3.27 | 22.00 ± 4.73 | 25.27 ± 1.96 | 25.80 ± 4.38 |
| WAIS-R VIQ | 87.83 ± 12.77 | 86.83 ± 11.50 | 88.60 ± 6.58 | 90.40 ± 6.80 |
| Digit Span* | 9.17 ± 2.29 | 6.67 ± 2.50 | 8.00 ± 2.12 | 8.20 ± 2.68 |
| Vocabulary | 8.17 ± 2.32 | 7.83 ± 1.72 | 8.40 ± 1.14 | 7.80 ± 1.10 |
| Arithmetic† | 7.50 ± 3.62 | 7.83 ± 2.93 | 6.40 ± 1.82 | 7.80 ± 2.59 |
| Comprehension | 6.25 ± 2.50 | 7.50 ± 2.07 | 8.20 ± 3.03 | 8.20 ± 1.92 |
| Similarities | 6.50 ± 2.07 | 7.17 ± 2.32 | 9.20 ± 2.18 | 9.00 ± 1.58 |
| GNT | 14.40 ± 6.83 | 15.83 ± 6.85 | 19.00 ± 2.55 | 18.25 ± 4.35 |
| VOSP Shape | 18.33 ± 1.17 | 18.83 ± 1.17 | 19.60 ± 0.55 | 19.60 ± 0.55 |
| VOSP Letters | 19.00 ± 0.89 | 19.00 ± 0.89 | 19.60 ± 0.55 | 19.60 ± 0.55 |
| RAVLT | | | | |
| – Trial 1 | 3.75 ± 0.96 | 3.80 ± 1.30 | 5.40 ± 2.07 | 5.20 ± 1.79 |
| – Trial 2 | 5.25 ± 1.89 | 4.60 ± 2.30 | 6.60 ± 2.07 | 7.20 ± 2.28 |
| – Trial 3 | 6.50 ± 1.29 | 5.60 ± 1.52 | 8.40 ± 1.82 | 8.60 ± 3.29 |
| – Trial 4 | 7.75 ± 1.89 | 6.20 ± 2.77 | 8.60 ± 2.51 | 9.20 ± 3.90 |
| – Trial 5 | 7.00 ± 0.82 | 7.00 ± 1.41 | 10.20 ± 3.70 | 9.60 ± 3.21 |
| Total | 30.25 ± 5.44 | 27.20 ± 8.26 | 39.20 ± 10.45 | 39.80 ± 13.94 |
| Delayed recall | 3.75 ± 1.26 | 5.00 ± 1.87 | 7.00 ± 4.47 | 6.00 ± 4.80 |
| Delayed recognition | 11.75 ± 1.26 | 11.00 ± 2.74 | 12.20 ± 3.49 | 13.20 ± 2.17 |
| RMF correct | 26.75 ± 2.40 | 33.41 ± 4.13 | 31.60 ± 2.07 | 34.20 ± 9.63 |
| SOPT4 errors | 0.25 ± 0.50 | 0.50 ± 0.83 | 0.20 ± 0.45 | 0.80 ± 0.45 |
| SOPT8 errors | 2.50 ± 0.58 | 1.50 ± 0.84 | 1.60 ± 0.55 | 1.60 ± 0.89 |
| SOPT12 errors | 3.75 ± 1.71 | 2.83 ± 0.75 | 3.20 ± 0.84 | 2.40 ± 0.89 |
| CET | 9.40 ± 6.37 | 10.50 ± 4.09 | 7.00 ± 1.87 | 9.00 ± 3.74 |
| RNG-RGI | 0.43 ± 0.07 | 0.41 ± 0.07 | 0.40 ± 0.05 | 0.41 ± 0.07 |
| RNG-REP | 3.20 ± 3.54 | 2.80 ± 2.63 | 1.00 ± 1.73 | 0.80 ± 0.84 |
| RNG-CS1 | 204.20 ± 97.95 | 164.00 ± 144.30 | 80.40 ± 55.34 | 87.00 ± 54.47 |
| RNG-CS2 | 72.77 ± 47.33 | 52.00 ± 10.28 | 64.80 ± 57.38 | 38.60 ± 8.08 |
| RNG-CST | 281.20 ± 128.63 | 201.20 ± 149.89 | 145.20 ± 58.50 | 125.60 ± 57.71 |
| RNG-GAP | 8.20 ± 0.75 | 8.00 ± 0.63 | 8.00 ± 0.71 | 7.20 ± 0.84 |
| VF* | 23.00 ± 12.90 | 15.00 ± 11.14 | 28.20 ± 9.36 | 28.4 ± 6.95 |

MMSE: Mini Mental State Examination; WAIS-R: Wechsler Adult Intelligence Scale-Revised; GNT: Graded Naming Test; VOSP: Visual and Object Space Perception Battery; RAVLT: Rey Auditory Verbal Learning Test; RMF: Recognition Memory for Faces; SOP: Self-Ordered Pointing Test; CET: Cognitive Estimates Test; RNG: Random number generation (see text for details of RNG measures) and VF: Verbal Fluency.
*indicates measures which showed significant deterioration from baseline to 3 months assessment in the surgery group but not the control group.
†indicates measures which showed significant improvement from baseline to 3 months assessment across the two groups.

sociated with a minimal risk of cognitive deterioration. Blumetti and Modesti's [6] sample of 10 patients the majority of whom ($n = 6$) had right-sided ventrolateral (VL) thalamotomy, included 5 with multiple sclerosis. Patients were assessed on the MMSE, the WAIS measure of intellectual ability, the Wechsler Memory Scale and the Halstead-Reitan Battery before and 12–24 months after surgery. No quantitative neuropsychological data were presented but it was reported that "receptive and expressive verbal performance as well as attending skills, were noted to deteriorate immediately after surgery relative to preoperative performance. This was noted in all cases with apparently more loss after left VL thalamic lesion but with significant improvement on these measures over time. Nonverbal memory impairment for both recognition and recall skills were noted more after right VL lesions with improvement over time". Given the 'significant improvement over time' noted by Blumetti and Modesti [6] it is difficult to establish what residual cognitive deficits remained at long-term follow-up, particularly in those with multiple sclerosis.

While our sample sizes were small, the two other previous studies evaluating the cognitive effects of thalamotomy in multiple sclerosis [6,46] only included 5 MS patients. Our study had the advantage of including a matched disease control group. Inclusion of the control group effectively controlled for any practice effects associated with repeated assessment, as evident in the improved performance on the Arithmetic subtest of the WAIS-R, as well as for the effects of disease progression or fluctuation during the 3 months follow-up period.

Both the current study and that of Schuurman et al. [46] found deficits in verbal fluency following thalamotomy in MS. In Parkinson's disease, a deficit in verbal fluency is the most consistent post-operative cognitive impairment [30,63] reported and has been previously documented following thalamic stimulation [46, 61], pallidotomy [22,44,47,52] as well as following pallidal or subthalamic nucleus stimulation [3,32,42, 48]. It is the only deficit that persists over time (18). Furthermore, there is some evidence of laterality effects, with verbal fluency found to be worse following left-sided surgery in some studies [22,44,46,52], but not others [4,47,57]. Given this consistently replicated finding that stereotactic surgery for PD or MS, whether thalamotomy, pallidotomy, or thalamic, pallidal, subthalamic stimuluation, has a negative impact on verbal fluency, the important question that remains to be addressed is the mechanisms of this adverse effect. Two main versions of the verbal fluency task are frequently used. For the phonemic verbal fluency, the participant is required to generate words beginning with a particular letter such as F, A, or S. In the categorical or semantic verbal fluency task, words belonging to a specific category, for example animals or furniture, are generated in one minute. Both versions of the verbal fluency task are attention-demanding and involve a number of internally controlled processes. For example, to generate words beginning with the letter F, patients have to actively search through associative networks and retrieve appropriate words (e.g. farm) while simultaneously suppressing production of highly associated but inappropriate words (e.g. cow), and monitoring of their output to prevent repetition of words already generated. In addition, during verbal fluency successful generation of words necessitates switching between phonemic (words beginning with fa... to fi... to fr..., etc.) or semantic (for example, when generating words from the category "animals" switching subcategories: birds, mammals, reptiles, farm animals, wild animals, domestic animals etc.,) categories [53,54]. In PD, the post-surgical impairment in verbal fluency is associated with deficits in switching between relevant subcategories [12,42]. In contrast to this reduction of switching (considered to be a 'prefrontal' function) during word fluency, the cluster size (considered to be a 'temporal' lobe function) is not altered following surgery for deep brain stimulation (DBS) of the STN [12,42]. It has been further noted that "The switching process relies primarily on the ability of set shifting, known to depend on the integrity of the prefrontal cortex" [12, p.293]. However, changes in switching can not be the sole mechanism responsible for the observed deficits in verbal fluency, since a number of studies have in fact shown that set-shifting or switching is improved with deep brain stimulation of the subthalamic nucleus [21, 29,32,62].

Imaging studies in healthy participants have established that verbal fluency involves activation of the dorsolateral prefrontal cortex and concomitant deactivation of the temporal cortex [15,16]. In PD, imaging suggests that verbal fluency deficits induced by stimulation of the subthalamic nucleus are associated with altered activation in the prefrontal-temporal networks engaged by this task [45]. It is possible that other forms of stereotactic surgery also disrupt the functioning of the fronto-temporal networks engaged by verbal fluency. This hypothesis needs to be tested in future studies.

Fatigue is a major component of the patients' experience of MS which could affect performance during neuropsychological assessment. Tests such as verbal fluency and digit span are attention-demanding and effortful. Ratings of fatigue and effort were not significantly altered from before to after surgery. This suggests that the changes in verbal fluency or digit span are not mediated by any alterations in fatigue or effort from before to after surgery.

Post-surgery, patients in the surgical group also performed worse on the Digit Span, a test which assesses attention and working memory, and on the MMSE, which is a measure of global cognitive functioning, with items assessing orientation, memory, executive function and language. In contrast, MS patients in the control group did not show any significant change on the digit span or MMSE tests from the first to the second assessment 3 months later. The MMSE results suggest that lesional sugery in the thalamic or subthalamic areas also affects attention, working memory and global cognitive functioning in MS. However, given that the other measures of working memory (Self-Ordered Pointing Test) or memory (Rey Auditory Verbal Learning Test, Recognition Memory for Faces) or language (Graded Naming Test) were not significantly altered by surgery, it is difficult to interpret the inconsistency of these results with the significant decline in Digit Span and MMSE scores. The Digit Span is sensitive to even momentary fluctuations of attention and patients can fail for this reason alone. Nevertheless, the decline in

MMSE scores in the surgical group was present for all six patients and this consistent decline suggests that it may be a reliable change. These results clearly require replication in a larger sample of MS patients undergoing stereotactic surgery who are followed-up over a longer period after surgery.
